# Identification of a suitable qPCR reference gene in metastatic clear cell renal cell carcinoma

**DOI:** 10.1007/s13277-014-2566-9

**Published:** 2014-09-16

**Authors:** Piotr M. Wierzbicki, Jakub Klacz, Agnieszka Rybarczyk, Tomasz Slebioda, Marcin Stanislawowski, Agata Wronska, Anna Kowalczyk, Marcin Matuszewski, Zbigniew Kmiec

**Affiliations:** 1 Department of Histology, Faculty of Medicine, Medical University of Gdansk, ul. Dębinki 1, PL 80-211 Gdańsk, Poland; 2 Department of Urology, Faculty of Medicine, Medical University of Gdansk, Gdańsk, Poland; 3 Department of Human Histology and Embryology, Faculty of Medical Sciences, University of Warmia and Mazury, Olsztyn, Poland

**Keywords:** ccRCC, Metastasis, Reference gene, *RPL13*, *GUSB*, Quantitative PCR

## Abstract

There is no data on reference gene (RG) selection in metastatic clear-cell renal cell carcinoma (mccRCC) for quantitative PCR (qPCR) data normalization. We aimed at selecting the most stable RG for further determination of new prognostic markers. Thirty-five nonmetastatic and 35 mccRCC patients undergoing radical nephrectomy were included. Paired primary tumor (T, *n* = 70) and normal (C, *n* = 70) kidney fragments were collected; from 12 out of 35 mccRCC cases, we also collected metastasized regional lymph nodes and adrenal gland tissues (M, *n* = 12). After RNA extraction, reverse transcription and qPCR were performed. Samples were divided into four analyzed groups. Fifteen candidate RGs were tested by RefFinder tool and manual statistics. To present the importance of RG selection, *TP53* gene expression levels in samples were normalized with the use of RG data. *RPL13* gene was the most stable RG in analysis of 35 primary tumor nonmetastatic versus 35 mccRCC samples and matched metastasized T/C/M samples (*n* = 12, each group). *GUSB* was the most suitable RG in total 152 samples and in paired T and C (*n* = 140) kidney samples. Expression of *GUSB*, *RPL13*, and the *RPL13* + *RPLP0* pair were independent of clinical/sample variables. Normalization of *TP53* expression levels showed variability of *GAPDH* and *ACTB* assays. *GUSB* or *RPL13* assays should be used in mccRCC for qPCR data normalization whereas *GAPDH* and *ACTB* assays should be avoided. Prior RG studies should precede each qPCR gene expression study since RG selection is associated with the origin and proportion of specimens.

**Electronic supplementary material:**

The online version of this article (doi:10.1007/s13277-014-2566-9) contains supplementary material, which is available to authorized users.

## Introduction

Renal cell carcinoma (RCC) is the third most common genitourinary malignancy, and its incidence has increased in the last 20 years [1]. The most frequent RCC subtype, clear-cell renal carcinoma (ccRCC) is characterized by a very high mortality rate of 40 %, due to distant metastases found in 30 % of RCC-diagnosed patients [2]. Although numerous prognostic RCC markers have been proposed (e.g., Ki67, TP53, CAIX) [3], there is an urgent need to perform gene expression studies of tumor/metastatic RCC in order to find new biomarkers [4]. Molecular analyses involve reverse transcription (RT), followed by quantitative PCR (qPCR), and the final expression results of target genes are based on normalization to any stably expressed internal reference gene (RG), measured in the same sample [5]. Expression level of a properly selected RG cannot be influenced by any clinical variable of the analyzed specimens/patients, i.e., sample origin, disease stage, or a pharmacological treatment [6, 7].

Most ccRCC gene expression studies are normalized to *GAPDH* or *ACTB* genes [6], whose variable expression levels were noticed in other malignancies [8–10]. Therefore, the first aim of our study was to select the most stable RG among 15 potential candidates in clinical material of primary nonmetastasic and metastasic tumor ccRCC matched with normal kidney tissue and ccRCC-origin metastasized tissues.

The second aim of the study was to analyze *TP53* gene expression rate with the use of obtained normalization data of all RGs in order to show that the gene expression results in ccRCC strongly depended on RG selection. The results of such molecular approach have not been published yet.

## Material and methods

### Patients and samples

**Fig. 1:**
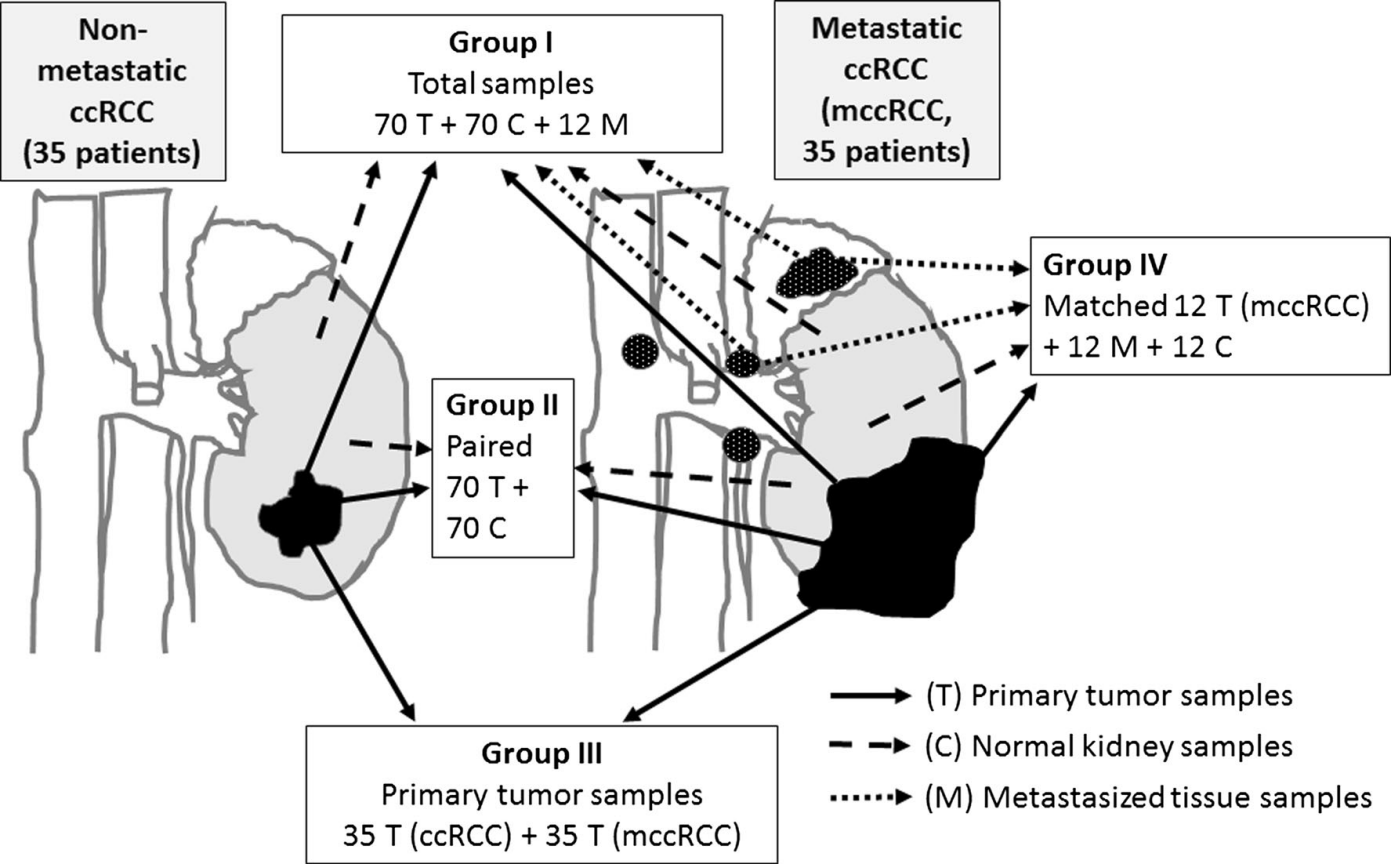
Sample origin and division into groups. Seventy patients undergoing radical nephrectomy were characterized by the local or distant metastases diagnosed in 35 cases (metastatic, mccRCC group) and 35 were nonmetastatic at the time of surgery. *Shaded color* represents normal kidney, *black pattern* represents primary tumor, and *white-dotted black pattern* shows regional metastasis to adrenal gland, paraaortic, and paracaval lymph nodes. Primary mccRCC tumors were characterized by the cancer spreading beyond Gerota’s fascia or into large veins (not shown to make figure lucid). From 12 mccRCC patients, the metastasized tissues were collected (*arrows* point example tissues), and together with matched primary tumor and normal kidney samples, group IV was created

Tissue samples were collected from 70 patients with ccRCC undergoing radical nephrectomy at the Department of Urology of the Medical University of Gdansk (MUG), Poland, between January 2011 and May 2013. The use of tissue material was approved by the Medical Ethical Committee of the MUG (decision no. NKEBN/4/2011), and informed written consent regarding the use of tissue was obtained before surgery from each ccRCC patient. One hundred fifty-two samples were classified into four groups as shown in Fig. 1. Thirty-five ccRCC cases did not show metastases at the time of nephrectomy whereas local and distant metastases were diagnosed in 35 ccRCC patients (metastasized ccRCC; mccRCC); five mccRCC cases showed distant metastasis: lung (*n* = 2), brain (*n* = 2), brain and liver (one patient) (Table 1). Other mccRCC patients possessed metastases located in regional paracaval or paraaortic lymph nodes. Metastases were also diagnosed in adrenal gland and vein thrombus. From all 70 patients, primary tumor (further named as “T”) and corresponding noncancerous fragments (named as “C”) of the kidney were obtained. Additionally, from 12 mccRCC patients, apart from tumor and kidney tissue, we also collected metastasized tissue samples (named “M”) from regional lymph nodes and adrenal glands (*n* = 10 and *n* = 2, respectively) due to additional lymphadenectomy and/or adrenectomy performed during nephrectomy (Fig. 1); metastasized tissue specimens from remaining 23 mccRCC cases were not obtained for this study. There was a positive correlation between TNM stage grouping and Fuhrman’s nuclear grades, *r*_*s*_ = 0.52, *P* < 0.001, Spearman’s test.

**Table 1:** Demographic and pathologic classification of ccRCC patients

Patients/specimens		Variable	Total samples, *n* = 70 (%)	Nonmetastatic ccRCC, *n* = 35 (%)	Metastatic ccRCC, *n* = 35 (%)	mccRCC cases with metastasized samples, *n* = 12 (%)
		Group division				
Age (years)^a^			60.97 ± 12.05	60.57 ± 13.41	61.37 ± 10.68	61.42 ± 6.86
Sex (M/F)			41/29	20/18	21/11	8/4
Tumor location		Left/right kidney	34/36	23/12	11/24	5/7
Fuhrman’s histological grade		1	4 (6)	4 (11.4)	–	–
		2	35 (50)	25 (71.2)	10 (28.5)	2 (17)
		3	19 (27)	4 (11.4)	15 (43)	8 (66.6)
		4	12 (17)	2 (6)	10 (28.5)	2 (17)
TNM stage grouping	Nonmetastatic	T1-2N0M0	30 (43)	30 (86)	–	–
		T2N0M0	5 (7)	5 (14)	–	–
	Metastatic	T1-2N1M0 T3N0-1M0	30 (43)	–	30 (86)	10 (83)
		T4N0-2M0T1-4N2M0 T1-4N0-2M1	5 (7)	–	5 (14)	2 (17)

^a^Data shown as mean ± SD

### Material acquisition

The dissected tissue samples of primary ccRCC tumor, normal kidney, and adrenal gland (ca. 7 ± 2 mm × 7 ± 2 mm × 7 ± 2 mm) or the whole lymph node (ca. 10 mm × 10 mm × 10 mm) were collected in the operating room no longer than 20 min after the kidney resection and placed in approximately five volumes of RNAlater (Ambion Inc., Austin, TX, USA). Three sectioned pieces of each sample were made. The central piece was used for RNA extraction, while the two side pieces were fixed in formalin and embedded in paraffin, followed by H&E staining and the examination performed by pathologist.

### RNA extraction and DNA digestion

Total RNA isolation was performed using GeneMATRIX Universal RNA Purification Kit (Eurx, Gdansk, Poland). Briefly, the tissues were homogenized in 2-ml tubes with ceramic beads (Blirt, Gdansk, Poland) in the presence of 300 μl lysis buffer (Eurx) in the MagnaLyser apparatus (Roche Diagnostics Deutschland GmbH, Mannheim, Germany) for 45 s at 6,000 rpm. Further processing was performed following the manufacturer’s (Eurx) protocol. Isolated RNA was eluted with 70 μl of nuclease-free water (Eurx), followed by quantification with spectrophotometer (Nanodrop ND 1000, Thermo Fisher Scientific, Fitchburg, WI, USA). The RNA integrity and quality were characterized by RNA integrity number (RIN) with the RNA 6000 Nano Kit using the Eukaryote Total RNA Nano Chip and Bioanalyzer 2100 apparatus (Agilent Technologies, Santa Clara, CA, USA). Next, 20 μl of extracted RNA was treated with TURBO DNA-free kit (Ambion) according to manufacturer’s protocol.

### First-strand cDNA synthesis

Complementary DNAs (cDNAs) were polymerized from 2 μg total RNA (100 ng RNA/1 μl RT reaction) of each sample using 0.5 μg oligo(dT)_18_ primers (Sigma-Aldrich, Munich, Germany), 200 U RevertAid Reverse Transcriptase, 1 mM dNTP mix, and 2 U Ribo-Lock (Fermentas-Thermo Fischer Scientific, Fitchburg, WI, USA). RT reaction was performed according to manufacturer’s protocol, and the resulting cDNA was stored at −25 °C after 10× dilution with nuclease-free water to be used as the template in qPCR analysis.

### Design and validation of reference gene primers

The primers were designed using Primer-BLAST software. The calibration curves for all gene-specific qPCR assays were performed (data not shown), and the resulting calibration curves’ data are presented in Table 2.

**Table 2:** Characteristics of candidate reference genes and *TP53* gene included in qPCR assays

Gene symbol	Gene name (common name)	Transcript ID	Primer sequence [5′ → 3′]	Slope (m)	*Y* intercept (b)^a^	PCR efficiency	Amplicon size (bp)	Intron spanning	Exons amplified	Annealing temperature (°C)
*ACTB*	Actin, beta (ß-actin)	NM_001101.3	CACAGAGCCTCGCCTTTGCC TGACCCATGCCCACCATCAC	−3.47	32.279	94.1	202	No	1–3	59
*B2M*	Beta-2-microglobulin	NM_004048.2	TTAGCTGTGCTCGCGCTACTCT TGGTTCACACGGCAGGCATACT	−3.504	31.111	92.9	294	No	1–2	59
*GAPDH*	Glyceraldehyd-3-phosphate dehydrogenase	NM_002046.4	CTGTTCGACAGTCAGCCGCATC GCGCCCAATACGACCAAATCCG	−3.333	31.804	99.6	110	Yes	1–3	59
*GUSB*	Glucuronidase, beta	NM_000181.3	ATGCAGGTGATGGAAGAAGTGGTG AGAGTTGCTCACAAAGGTCACAGG	−3.33	38.685	99.7	177	No	8–9	57
*HMBS* (*PBGD*)	Hydroxymethylbilane synthase (porphobilinogen deaminase)	NM_000190.3	CACCCACACACAGCCTACTTTCC GTGAACAACCAGGTCCACTTCATTC	−3.303	44.164	100.8	318	Yes	1–6	58
*HPRT1*	Hypoxanthine-phosphoribosyl-transferase 1	NM_000194.2	GACTTTGCTTTCCTTGGTCAGGC TGGCGATGTCAATAGGACTCCAG	−3.03	38.91	100.7	279	No	6–9	57
*IPO8*	Importin 8	NM_006390.3	TTGGAAGAAACCGCGCTTGAGG ACCAGGCTGCATCTCGACTCTG	−3.308	45.335	100.5	118	No	23–24	59
*PGK1*	Phosphoglycerate kinase-1	NM_000291.3	GTTGACCGAATCACCGACCTCTC AGAACAGAACATCCTTGCCCAGC	−3.48	35.561	93.2	329	Yes	1–4	58
*PPIA* (*CYPA*)	Peptidyl-prolyl isomerase (cyclophilin A)	NM_021130.3	CTTGGGCCGCGTCTCCTTTGAG GCTTGCCATCCAACCACTCAGTC	−3.512	36.133	92.6	329	Yes	1–5	59
*RPL13*	Ribosomal protein L13	NM_000977.3	TTCCGCTCGGCTGTTTTCCTG GGGCCTTACGTCTGCGGATCTTA	−3.325	39.714	99.9	164	Yes	1–2	59
*RPL32*	Ribosomal protein L32	NM_000994.3	GCGCCACCGTCCCTTCTCTC GACATATCGGTCTGACTGGTGCC	−3.372	43.363	97.9	155	No	1–3	58
*RPLP0*	Ribosomal protein, large, P0	NM_001002.3	TAAACCCTGCGTGGCAATCCCTG TGAACACAAAGCCCACATTCCCC	−3.422	32.553	95.9	307	No	2–4	58.5
*TBP*	TATA box-binding protein	NM_001172085.1	GCTGTTTAACTTCGCTTCCGCTG GGTGTTCTGAATAGGCTGTGGGG	−3.484	39.408	93.7	135	No	3–5	58
*TFRC*	Transferrin receptor (p90, CD71)	NM_001128148.1	GAGGACGCGCTAGTGTTCTTCTG GTTCCTGCCAGTCTCTCACACTC	−3.627	45.757	88.7	348	Yes	2–4	58
*UBC*	Ubiquitin C	NM_021009.5	ACTGGTTTTCTTTCCAGAGAGCGG GGAGGGATGCCTTCCTTATCTTGG	−3.344	47.163	99.3	309	No	1–2	59
*TP53*	Tumor protein p53, isoform a	NM_001126112.2	ACGACGGTGACACGCTTCCCTG CGCTAGGATCTGACTGCGGCTC	−3.52	44.121	92.36	84	No	1–2a	60

^a^ *Y* intercept indicates the expected Ct for a sample with copy number per 1 pg of total RNA for reverse transcription

The selection of RG assays for this study was based on the following: MeSH database search for the most commonly used RGs in ccRCC and in other cancers; previous literature results of normalization studies of kidney and other cancers [6, 10–14] and the commercially available RG sets (Roche Diagnostics, SA Biosciences, Life Technologies/Applied Biosystems).

For the RGs assays, the 15-μl reaction mixture included 1.5 μl 10× diluted sample cDNA, 0.2 μM each forward and reverse primers and SensiFast Sybr™ No-Rox kit (Bioline, London, UK). qPCR reactions for all RGs were performed in triplicate in StepOne Plus apparatus (Life Technologies/Applied Biosystems, Grand Island, NY, USA), and the geometric mean of cycle threshold (Ct) values was used in further analyses with StepOne Software ver. 2.2 (Life Technologies/Applied Biosystems). We set runs for each gene assay separately. Each qPCR run contained paired T/C or T/C/M samples, no-template control (water instead of cDNA), and 10× diluted pooled cDNA as a run-to-run precision control.

Intra-run and inter-run precision control tests were set using 10× diluted pooled cDNA in the same conditions as routine RGs assays. The intra-batch control was based on qPCR reactions for *GUSB* assay in 15 replicates during the same run. Inter-batch (run-to-run) test was based on data obtained from separate runs (*n* = 5) as described above, for each RG assay separately. In both tests, raw Ct data were collected and mean ± standard deviation (SD) values were computed. Ct coefficient of variation (CtCV%) values were calculated using the following formula: CtCV = SD/mean × 100 %.

The gene *TP53* which was targeted for normalization in ccRCC was amplified using the same conditions as RGs (details in Table 2).

Immunohistochemistry (IHC) methodology for TP53 protein is presented in supplementary materials.

## Data analysis

### Statistical analysis

Raw Ct data was transferred and calculated using GraphPad Prism, ver. 6.05 (GraphPad Software, San Diego, CA, USA). Normality of qPCR and clinical data was checked using D’ Agostino and Pearson omnibus test. Nonparametric Mann-Whitney *U* (A) and Kruskal-Wallis ANOVA tests (B) were used to compare clinical and qPCR data.

#### Selection of single reference gene with the use of specialized tools

The free online software tool, RefFinder [15] was applied, which uses the following algorithms: BestKeeper [16], NormFinder [17], GeNorm [7], and the relative delta Ct (dCt) [18] method. The final ranking from 1 (best RG) to 15 (least suitable RG) was shown as a result.

#### Selection of RG pair

GeNorm data for a pair of RG was transferred from RefFinder results. For NormFinder RG pair selection, raw Ct data were calculated into arbitrary units (AU) with the use of calibration curves data (Table 2).

#### Association of RG expression and clinical data

Raw Ct qPCR data of RG assays were compared with different clinical variables (Table 1) with the use of statistical tests described above (A or B).

#### RGs expression level versus sample origin

Raw Ct data for each RG assay were divided according to the sample origin (T, C, or M) and analyzed group (I–IV); statistical association between samples’ Ct values were assessed using A or B tests.

#### Gene normalization based on RG data

The expression of *TP53* was calculated using raw Ct data normalized with the use of Ct data of each RG. Further, expression data of *TP53* in control samples was set to 1 and the expression of *TP53* in T and M samples were calibrated to results obtained in control samples.

## Results

### RNA quality control

The average RNA concentration was 379.51 ± 222.21 ng/μl (range 76.00–1,980.60 ng/μl). All samples presented high RNA quality and integrity; mean A_260/280_ ratio was 2.02 ± 0.07 (range 1.96–2.13); average RIN values from all samples were 8.32 ± 1.12 (range 5.5–9.8).

### Intra- and inter-batch qPCR precision control

As a part of a validation procedure of the presented qPCR method [19–21], pooled cDNA (10× diluted) from all samples was used as precision control material for each RG assay run. For intra-batch precision test mean Ct ± SD was 25.12 ± 0.247; therefore, CtCV% value was 0.98 % for *GUSB* assay (Supplementary Fig. 1). The inter-batch precision values of CtCV% ranged from 0.37 % for *GUSB* to 0.74 % for *HMBS* (details not shown). Obtained results fulfill the precision criteria of bioanalytical method validation (CtCV% < 15 %) [19, 22].

### Selection of a single RG

**Fig. 2:**
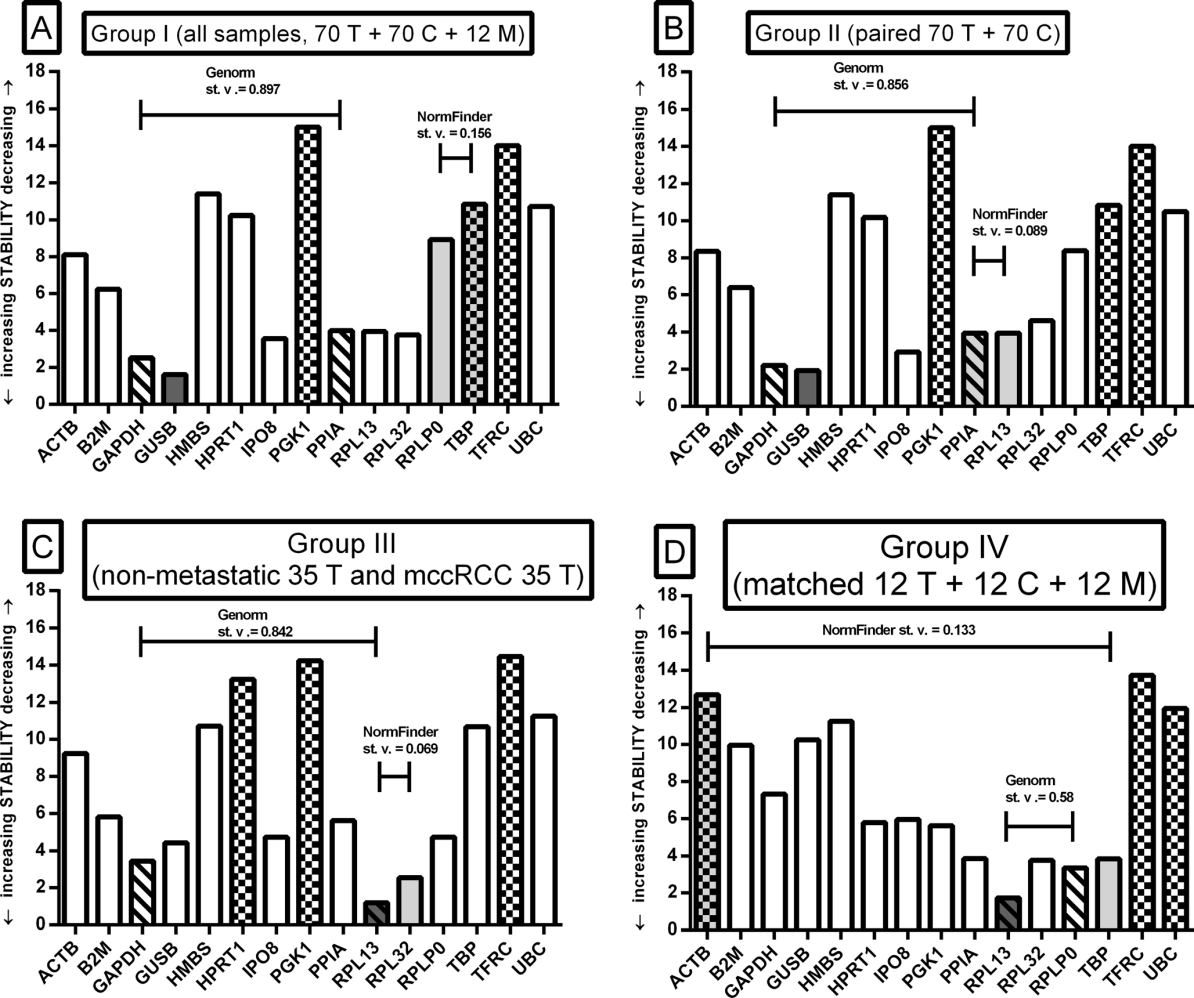
Summary selection of best single RG or RG pair with the use of RefFinder, GeNorm, and NormFinder. **a** All samples (*n* = 152), **b** paired primary tumor and control kidney samples (*n* = 140), **c** primary nonmetastatic and mccRCC tumor samples (*n* = 70), **d** metastatic group with matched tumor, normal, and metastatic samples (*n* = 36). *Dark gray bar* shows the best single RG whereas *chequered pattern* shows the least stable genes in the analysis. *Light gray bars* depict RG pair selected by NormFinder (with stability value of the pair), while *left diagonal bars* represent GeNorm RG pair (with stability value of the pair)

Raw Ct data of all RGs assays (Supplementary Fig. 2) were analyzed by RefFinder [15]. The results for analyzed groups of samples (Fig. 1) are shown in Fig. 2. Since RefFinder utilizes summary results obtained from dCt, BestKeeper, GeNorm, and NormFinder, a brief presentation of each tool with results are described below.

#### Relative delta Ct

dCt method was applied by Silver et al. [18] and is based on the mean SD value calculated between Ct data of each tested RG in comparison to other assays (e.g., *GUSB* vs *ACTB*, followed by *GUSB* vs *GAPDH*); RG assay with the smallest SD number is judged as the most stable. *GUSB* was the most stable RG in group I (all samples), *GAPDH* in group II (paired 70 T + 70 C), followed by *RPL13* in groups III (35 nonmetastatic vs 35 mccRCC) and IV (matched tumor-metastasized control group of samples).

#### BestKeeper

The tool utilizes statistical calculations of each RG assay separately; RG with both smallest SD and Pearson’s correlation coefficient values is considered as the most stable [16]. BestKeeper ranked *GUSB* as the most stable in groups I and II, whereas *RPL13* and *RPL32* were selected as best RGs in groups III and IV, respectively (Supplementary Table 1).

#### NormFinder

NormFinder utilizes a special algorithm which combines group division, absolute copy number of gene, and variation caused by biological and experimental factors [17]. Therefore, raw Ct data had to be computed into AU by our team (see “Materials and methods” section) before NormFinder analysis. This algorithm selected *GUSB* as the best single RG in groups I and II and *RPL32* and *RPL13* in groups III and IV, respectively. NormFinder’s RGs pair selection are presented further.

#### GeNorm

GeNorm calculates a normalization factor (*M*) based on a geometric mean of Ct values of at least two RGs. Stepwise exclusion of the least stable gene allowed the genes to be ranked according to the stability value (the lower the M value, the higher the gene’s expression stability) [7]. RefFinder’s built-in GeNorm algorithm chose a combination of two candidate RGs with the lowest *M* values, and the results are shown in Fig. 2.

#### RefFinder summary ranking of a single RG

RefFinder, free online tool, combines results of the mentioned single-RG algorithms. Besides RefFinder’s availability and simplicity (copy-paste of raw Ct data staged in columns), the more RGs are tested, the more precise result is obtained. Figure 2 and Supplementary Table 1 show RefFinder ranking; *GUSB* was selected as the best RG in groups I and II, and *RPL13* was the most suitable gene in groups III and IV.

### Selection of RG pair

Due to recommendation to use at least two normalizers for gene expression analysis instead of one [5, 20], GeNorm and NormFinder results were applied. Due to differences in algorithms, no common scores between tools for any analyzed group of tissues (linked RGs in Fig. 2) was observed (with one exception; *GAPDH* + *PPIA* was selected by GeNorm as best pair in groups I and II). As a result we selected seven different RG pairs in four sample groups. In order to clarify this disagreement, we found that the CtCV% factor has been recommended to validate NormFinder and GeNorm results [23]. We checked CtCV% values for all selected RG pairs together with single RGs in different groups (Supplementary Table 2) and observed that NormFinder’s selections were characterized by lower CtCV% values than GeNorm’s choices. Although such result shows that RGs pairs selected by NormFinder are more stable than GeNorm’s pairs, we also found that for groups I–II, the best single RG showed lower CtCV% value than for any RG pairs (6.58 and 6.19 % for *GUSB* vs 8.21 and 8.36 % for NormFinder’s pair for groups I and II, respectively, Supplementary Table 2). Moreover, single RG for groups III and IV—*RPL13* presents CtCV% value between pairs of NormFinder and GeNorm. Based on those results, we suppose that despite RG pairs selected by NormFinder better meet the statistical requirement than GeNorm’s, they do not outdistance single RG selection.

### Relationship of RGs expression and experimental variables

Apart from selection of the most stable gene in selected groups with the use of specialized tools, the final result of RG selection has to be supported by manual statistics which utilize clinical variables and material division into subgroups according to their origin [6, 10, 14]. Only genes which are completely independent of those variables (in statistical perspective; *P* > 0.05 between subgroups) can be finally chosen as a suitable RG in selected group of samples.

#### Association between RGs level and clinical status of ccRCC patients

Single RGs qPCR data of all 152 samples (Supplementary Fig. 2) were compared to clinical data of the ccRCC patients (Table 1). We also added comparison of RG pairs selected by GeNorm or NormFinder algorithms; the statistical results are shown in Table 3. The expression of any genes or RG pairs was not associated with gender and age of patients. Interestingly, we observed that *GAPDH*, *GUSB*, *IPO8*, *RPL13*, *RPL32*, and *UBC* genes, as well as *RPLP0* + *TBP*, *RPL13* + *RPL32*, *RPL13* + *RPLP0*, and *ACTB* + *TBP* RG pairs, were the only assays whose expression did not depend (*P* > 0.05) on cancer progression. Since the expression levels of remaining RGs were associated with at least one selected clinical factor, they should not be further included in normalization panel of ccRCC studies (Table 3).

**Table 3:** Statistical relationship between data of ccRCC patients and qPCR data of single reference genes and RG pairs

Gene pair	Sex	Age	TNM stages	Nonmetastatic vs metastatic ccRCC (TNM stages grouped)	Fuhrman’s histological grade (1–4)
*ACTB*	ns	ns	ns	ns	0.04
*B2M*	ns	ns	ns	ns	0.0026
*GAPDH*	ns	ns	ns	ns	ns
*GUSB*	ns	ns	ns	ns	ns
*HMBS*	ns	ns	0.0066	0.033	ns
*HPRT1*	ns	ns	ns	ns	0.002
*IPO8*	ns	ns	ns	ns	ns
*PGK1*	ns	ns	<0.0001	0.001	0.0075
*PPIA*	ns	ns	ns	ns	0.003
*RPL13*	ns	ns	ns	ns	ns
*RPL32*	ns	ns	ns	ns	ns
*RPLP0*	ns	ns	0.0046	0.002	ns
*TBP*	ns	ns	0.0001	0.0029	0.0003
*TFRC*	ns	ns	ns	ns	0.0026
*UBC*	ns	ns	ns	ns	ns
*GAPDH* + *PPIA* ^a^	ns	ns	0.0011	0.0011	ns
*RPLP0* + *TBP* ^b^	ns	ns	ns	ns	ns
*PPIA* + *RPL13* ^b^	ns	ns	0.0012	0.0005	0.02
*GAPDH* + *RPL13* ^a^	ns	ns	0.0245	0.011	ns
*RPL13* + *RPL32* ^b^	ns	ns	ns	ns	ns
*RPL13* + *RPLP0* ^a^	ns	ns	ns	ns	ns
*ACTB* + *TBP* ^b^	ns	ns	ns	ns	ns

Ct data was calculated as geometric mean of each assay. Statistical tests used: Mann-Whitney *U* test—sex, nonmetastatic (T1-2N0M0 + T2N0M0) versus metastatic (T1-2N1M0, T3N0-1M0 + T4N0-2M0, T1-4N2M0, T1-4N0-2M1) ccRCC; Kruskal-Wallis ANOVA—age, TNM stage, Furhman’s histological grade (1–4). *P* < 0.05. ns: *P* > 0.05. Ct data were used for statistical comparisons

^a^RG pair as selected by GeNorm for groups I–IV

^b^RG pair as selected by NormFinder for groups I–IV

#### Analysis of RG expression in subset of biopsies

**Fig. 3:**
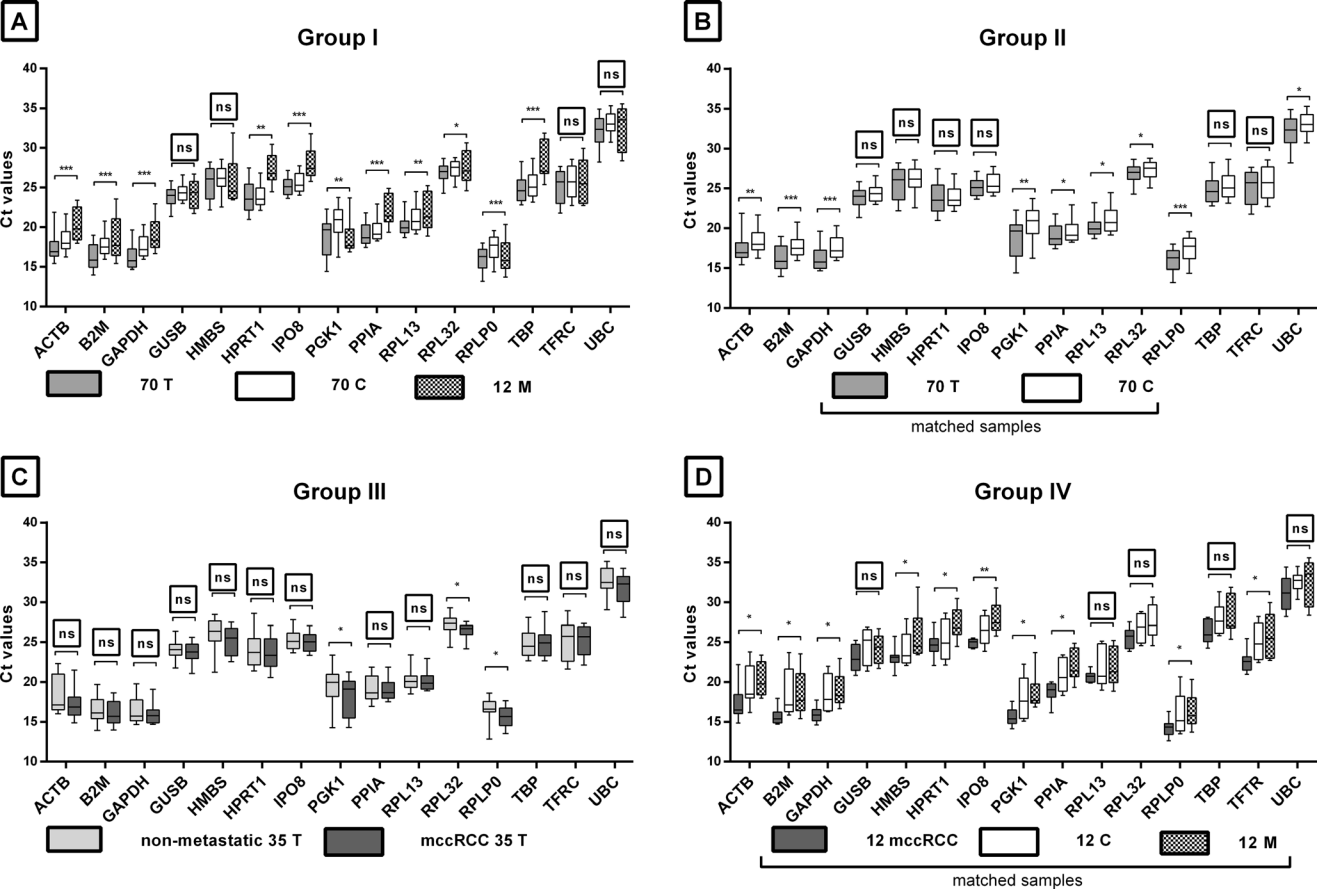
Expression levels of candidate reference gene assays in analyzed groups divided by sample origin. Summary results of all RG assays (*x* axis) and Ct data (*y* axis). *Box* (median Ct and lower and upper quartiles) and *whisker* (10–90 % Ct values) plot for each samples’ subgroup. **a** All samples (*n* = 152), **b** paired primary tumor and control kidney samples (*n* = 140), **c** primary nonmetastatic and mccRCC tumor samples (*n* = 70), **d** metastatic group with matched tumor, normal, and metastatic samples (*n* = 36). Statistics used: **a**, **d**—Kruskal-Wallis ANOVA; **b**, **c**—Mann-Whitney *U* test. *0.01 < *P* < 0.05; **0.001 < *P* < 0.01; ***P < 0.001; *ns*, *P* > 0.05 between subgroups

Expression data of each single RG in 152 samples were divided into groups I–IV, followed by statistical comparison according to their origin; e.g., expression levels of 70 T versus 70 C for group II. Summary plots are presented in Fig. 3. We observed that the higher the sample heterogeneity occurs in analyzed group, the fewer the genes share similar (origin-independent; *P* > 0.05) expression pattern between subgroups; if 70 T versus 70 C (group II) were assessed, expression pattern of six genes was comparable (Fig. 3b, *P* > 0.05) in comparison to group I with only four genes when 12 M subgroup was introduced. We therefore observed the highest similarity between RG expression levels between samples when nonmetastatic versus metastatic tumor specimens were compared (group III, Fig. 3c). As a result, candidate genes which passed this statistical test can be considered in final RG result in selected groups of samples.

**Fig. 4:**
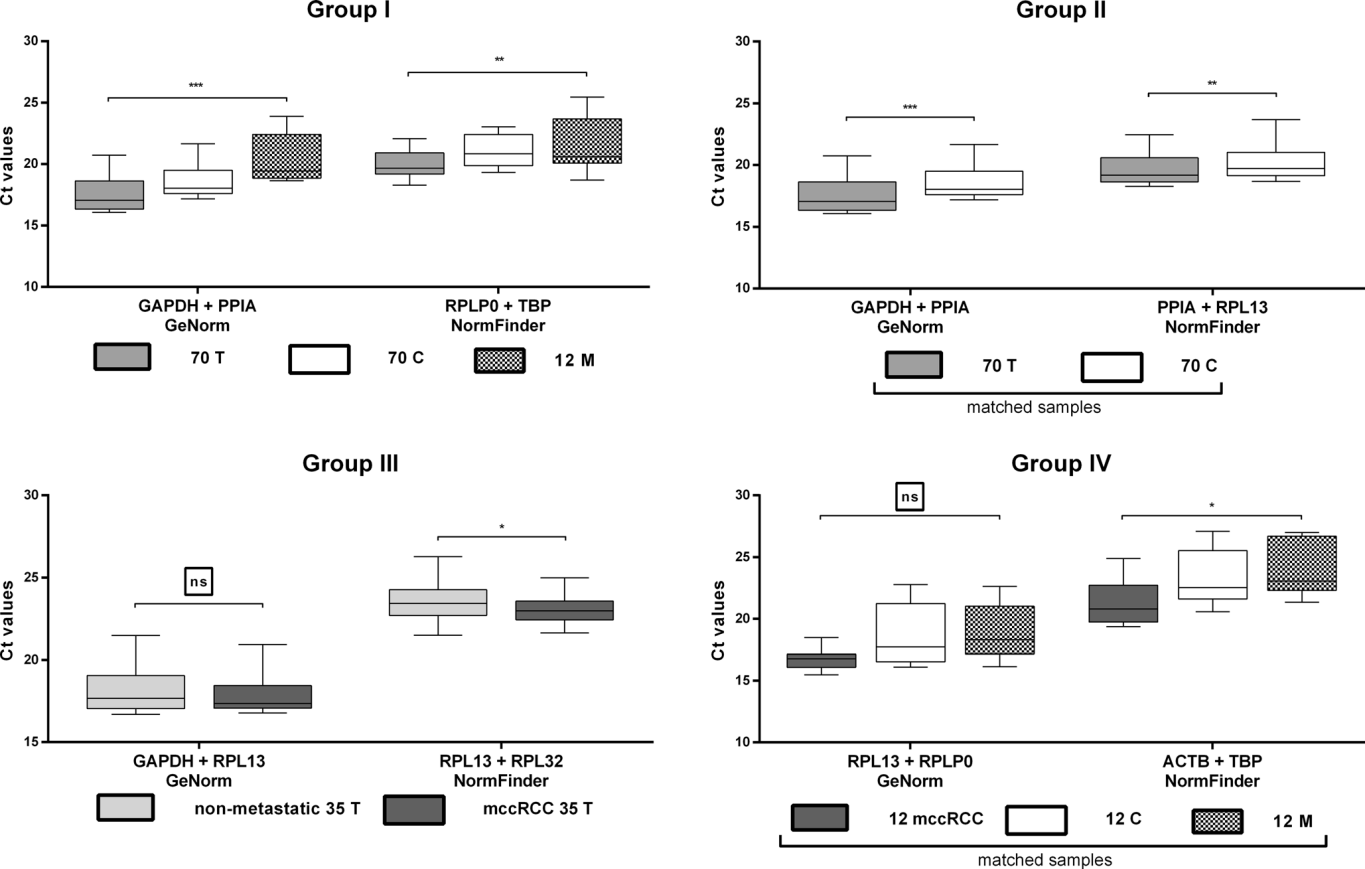
Expression levels or candidate reference gene pairs in specimens’ subgroups. RG pairs were selected by either GeNorm or NormFinder algorithms, separately for each analyzed group. Ct data were calculated using geometric mean of Ct score for each gene. Figure legends according to Fig. 3

Comparable statistical analyses were performed for seven RG pairs selected by GeNorm and NormFinder (Fig. 4). We observed that only *GAPDH* + *RPL13* and *RPL13 + RPLP0* pairs share similar expression levels in groups III and IV, respectively; therefore, other RG pair should be omitted from the final selection.

### Impact of reference gene on the relative expression of *TP53* gene

**Fig. 5:**
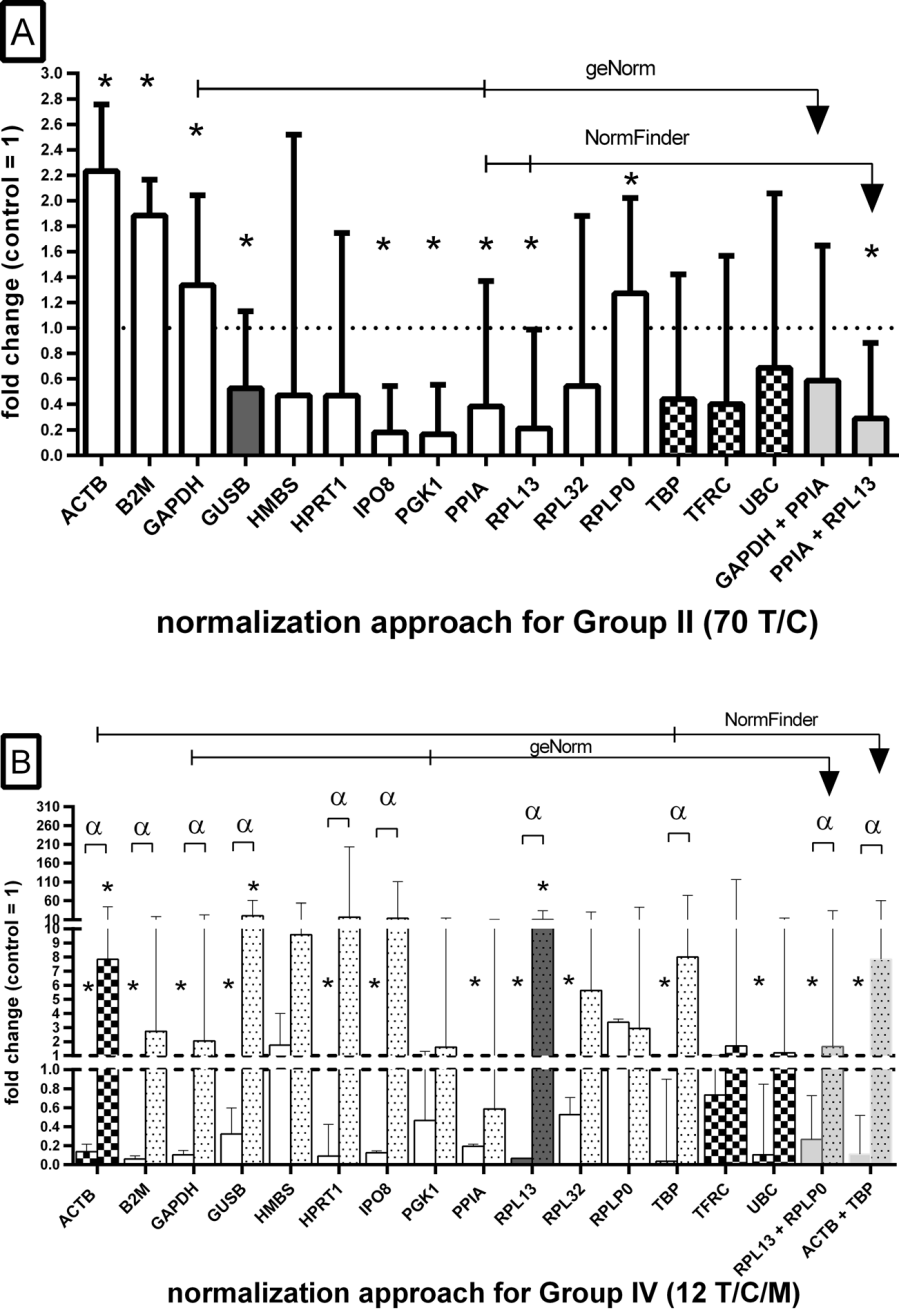
Normalization of *TP53* gene expression with the use of RG data. In selected groups II and IV, the expression of *TP53* for control samples (70 and 12 C) were set to 1 and expression values in the matched tumor (70 T) or tumor and metastasis (12 T/M) were calculated as multiplies. The *columns* represent the median and interquartile ranges of the *TP53* gene expression. *Dark gray bars* represent the most stable single RG selected for the analyzed specimens’ group; *chequered pattern* shows the least stable genes in the analysis, according to RefFinder results. *Light gray bars* show the relative expression normalized to geometric mean of RG pairs selected by GeNorm and NormFinder algorithms, respectively. **a** Relative expression in paired 70 T samples in relation to 70 C specimens (expression set to 1). **b** Relative expression in matched 12 T (*blank*) and 12 M (*dotted boxes*) in relation to 12 C specimens. *P < 0.05 between T or M samples and control (Wilcoxon test), α*P* < 0.05 between matched T and M samples (Wilcoxon test). Please note that results for 70 T (**a**) and 12 M (**b**) cannot be directly compared due to different control groups

The expression analysis of the target gene *TP53* were used in this study to demonstrate the effect of different normalization genes on the relative expression data (Fig. 5a, b). For paired tumor and normal samples (group II), we observed increased expression of *TP53* if *ACTB*, *B2M*, *GAPDH*, or *RPLP0* were used for normalization. We also observed that for the least suitable RGs (chequered bars in Fig. 5a), there were no statistically significant differences between T and C samples.

When the matched 12 T/C/M samples were investigated (Fig. 5b), the expression pattern of *TP53* was different than for the 70 T/C group, i.e., we observed decreased *TP53* expression in T samples for 11 of 15 RGs, including *ACTB* and *GAPDH*, as well as when GeNorm and NormFinder RG pairs were used for normalization (*P* < 0.05, Fig. 5b).

Based on a well-documented decreased expression pattern of *TP53* gene in ccRCC tumor samples [24, 25], we decided to apply candidate RGs which showed significant under expression of *TP53* in tumor samples in both normalization approaches (group II and group IV). Therefore, only *GUSB*, *IPO8*, *PPIA*, and *RPL13* genes, as well as *PPIA* + *RPL13*, *RPL13* + *RPLP0*, and *ACTB* + *TBP* pairs, passed this test. The different levels of *TP53* expression for *ACTB*, *GAPDH*, and *B2M* in relation to number of analyzed cases should exclude those genes from RG panel for ccRCC, since the expression results of *TP53* cannot change by the increased number of analyzed samples (i.e., under expression of *TP53* when 12 T/C were selected versus overexpression of *TP53* when 70 T/C was analyzed). We also observed only small increase of *TP53* expression (ca. 2× increase vs control) in metastasized samples when *GAPDH* was utilized for normalization in contrast to 10× higher expression when *RPL13* was used as RG (Fig. 5b).

**Fig. 6:**
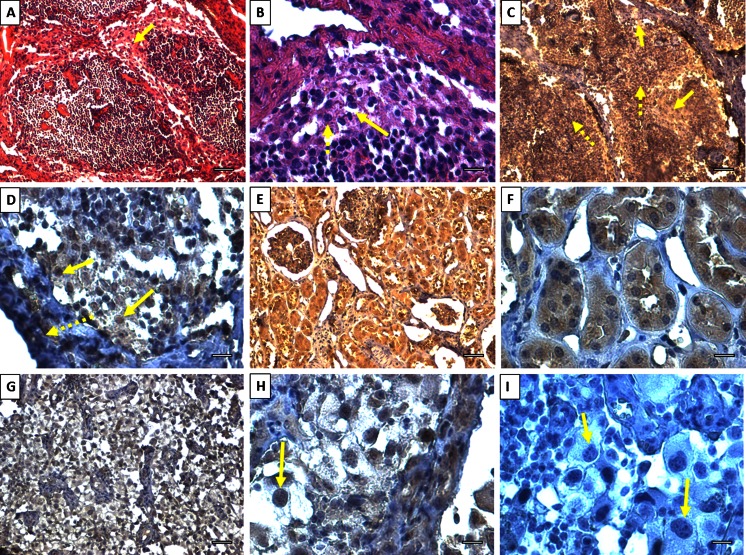
Representative histological and immunohistochemical pictures of ccRCC patient’s tissue (male, 73 years, Fuhrman grade 4, pT3aN1Mx). Hematoxylin and eosin staining of ccRCC metastasis to perirenal lymph node **a** ×100, **b** ×400). *Arrow* shows metastasized cells with clear-cell and eosinophilic RCC pattern (*dotted arrow*). **c**, **d** IHC staining for TP53 protein presents high accumulation of protein in cytoplasm of immune cells (*dotted arrow*) and lower accumulation in cytoplasm of metastasized cells (*solid arrows*). **d** The presence of TP53 is limited mostly to nuclei of malignant cells; no TP53 presence in lymph node trabeculae (*dotted arrow*). **e**, **f** Normal kidney’s cortex fragment stained for TP53; the very high presence of TP53 is found in the cytoplasm of podocytes and tubular cells. **g**, **h** IHC staining for TP53 in tumor ccRCC; the protein is limited mostly to nuclei of malignant cells. **i** Negative control (without primary Ab, only hematoxylin staining) for TP53 in ccRCC (×400); obviously big and irregular shape of nuclei with up to five visible nucleoli indicate Furhman’s grade 4. Please note that high accumulation of TP53 in **h** obscures the nuclei details found in **i**

Additionally, we checked the presence of TP53 protein in matched biopsies of T/M/C samples (group IV) with the use of immunohistochemistry (IHC, supplementary methods). IHC staining showed high expression of TP53 in normal kidney, with its dispersed distribution in the cytoplasm of epithelial cells. On the contrary, in tumor ccRCC slides, we observed a high accumulation of TP53 mainly in the nuclei of malignant cells (Fig. 6e–h). Moreover, we observed massive presence of TP53 protein in all metastasized and normal immune cells of lymph nodes (Fig. 6c, d), which was in accordance with upregulation of *TP53* mRNA (Fig. 5b) in metastasized samples.

### Reference gene final selection

Based on single reference gene selection (RefFinder) together with exclusion results of clinical/biological versus RG expression statistical tests, as well as *TP53* normalization approach, we found that *GUSB* is the most suitable single RG for groups I and II and *RPL13* is best RG for groups III and IV (Table 4). Different pattern occurs for RG pair selection, since only *RPL13* + *RPLP0* selected by the GeNorm algorithm (Table 5) fulfils statistical criteria; therefore, for matched tumor-control-metastasized ccRCC samples (group IV) *RPL13* + *RPLP0* pair may be considered for normalization in gene expression studies.

**Table 4:**
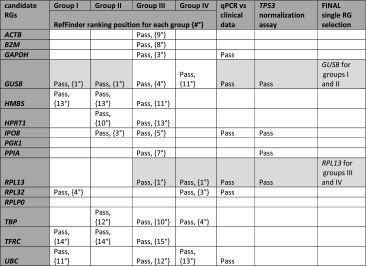
Final selection of a single RG in ccRCC groups based on summary data of normalization algorithm and manual statistics

Pass—expression level of RG was nonsignificantly different between sample subgroups (*P* > 0.05) in group division; independent of some clinical factor (*P* > 0.05); or TP53 was statistically decreased (*P* < 0.05) in tumor samples when normalized to selected RG. Empty cells—RGs which did not fulfill the mentioned statistical criteria. Pale gray cells—the most suitable and common scores for specific RG

**Table 5:**
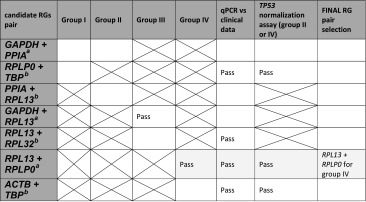
Final selection of RG pairs based on NormFinder or GeNorm results followed by manual statistics

Pass—expression level of RGs pair was nonsignificantly different between sample subgroups (*P* > 0.05) in group division; independent of some clinical factor (*P* > 0.05); or TP53 was statistically decreased (*P* < 0.05) in tumor samples when normalized to selected RGs pair. Empty cells—RGs which did not fulfill the mentioned statistical criteria. X—test was not assessed for a selected pair. Pale gray cells—the most suitable and common scores for specific RGs pair

^a^GeNorm RG pair selections for different groups

^b^NormFinder RG pair selections for different groups

## Discussion

In this study, we presented methods and results of selection of suitable RGs in metastatic ccRCC, because currently, there is no data concerning this important issue. A reliable normalization project should be based on validation procedures related to analytical biochemistry [6, 19]. Since the presented data contain standardization of sample preparation and RNA integrity assessment, followed by qPCR requirements [5, 20]—standard curve, precision tests (intra- and inter-run), and selectivity (melt curve)—we believe that our methodology and results can be utilized by other researchers.

To date, only three groups performed similar RG selection studies on tumor ccRCC compared to normal kidney tissue [6, 26, 27], while Bjerregaard et al. focused on ccRCC versus renal oncocytoma [10]. When compared with the mentioned data, our study was based on nonmetastatic versus mccRCC cases and analyzed the highest number of RGs. Although RGs’ suitability was calculated and ranked with the use of specialized tools, the limitation of those algorithms is the utilization of raw expression data without consideration for either clinical data or sample’s primary localization. Therefore, the statistical comparison of clinical or specimen’s origin versus expression data had to be evaluated with the use of manual statistics, since one of the basic requirement for RG selection is the independence of expression from biological and clinical variables [6, 7]. We therefore observed the independence of suitable RGs’ expression from clinical factors, which was in accordance with other authors’ observations [6, 27, 28].

Our results of RG selection are not in agreement with other authors who focused on RCC. Although Jung et al. found *TBP* + *PPIA* as the most suitable RG pair, their study on 25 cases introduced 24/1 male/female (M/F) and no patients with diagnosed metastasis, whereas our project contained larger population sample (41/29 M/F) and 35 mccRCC (T3 or N1-2 or M1) cases. The most recent report on RG selection in ccRCC shows that *PPIA* and *RPS13* (or their combination) is based on paired tumor control kidney specimens (no metastasized tissue) from 16 (14/2 M/F) ccRCC patients [27]. Furthermore, only 5 of 16 patients from the Dupasquier et al. study presented local or distant metastases [27]. Finally, in contrast to utilization of the same algorithms and manual statistics, ours and the mentioned studies data cannot be strictly compared, since they did not check stability of *GUSB* or *RPL13* genes [6, 27].

The limitation of our study is, however, the lack of distant metastasized samples (bone, lung) and utilization of only 12 samples obtained from regional metastases. Therefore, our findings should be confirmed by independent studies performed by other teams.

Our results also contained the selection of RGs pair. Although the use of two or more RGs in gene expression studies has been strongly recommended by minimum information for publication of quantitative real-time PCR experiments (MIQE) guidelines [20], cancer gene expression studies rarely utilize more than one RG for the normalization [29–31]. In fact, the most recent review which focused on observance of MIQE in qPCR gene expression studies in colorectal cancer revealed that only 6 of 179 (3.3 %) analyzed studies (between 2006 and 2013) used two RGs [21]. Therefore, we do not suppose that the requirement for two or more RGs in gene expression studies is also followed in ccRCC; hence, we propose to use one but carefully selected RG. Additionally, despite the fact that GeNorm tool offers the evaluation of a number of RGs recommended for normalization (two or more RGs), we did not find any reference which uses three or more RGs in cancer gene expression studies. Finally, since no common results were found between individual algorithms or groups’ divisions, followed by statistical failure of most selected RGs pairs, we propose to focus on single-RG normalization.

We chose to create different analyzed groups of samples and presented complex results based on this division. It was connected to the fact that we utilized a large number of specimens; therefore, we decided to present as much useful data as possible. Although the presentation of more than only one comparison (nonmetastatic vs metastatic tumor samples) [14] could be obscured, our results for more groups can be easily utilized by other teams working on different origin of samples, e.g., paired tumor control samples for studies of ccRCC cancer development markers.

Our normalization approach of *TP53* gene expression has clearly shown that the choice of an appropriate reference gene can strongly affect final results of gene expression studies. We chose the *TP53* tumor suppressor gene, whose decreased expression in tumor samples of ccRCC and other malignancies was proven by other studies [24, 25, 32, 33]. Apart from other presented statistical results in this study, we propose to exclude *GAPDH* and *ACTB* from the normalization panel, since false results can be obtained when normalized to those RGs. Although *GAPDH* is the most common RG in RCC studies and most author may disagree with our results, *GAPDH* expression stability should also be questioned when the biological characteristics of RCC is taken into consideration. Since tumor RCC is characterized by dysregulation of glucose metabolism [34], it is a strong support for nonconstant expression of *GAPDH* [23]. Moreover, it was noted that GAPDH protein level changes in RCC as well in other cancers [9]. Our results of *TP53* normalization in metastasized group with the use of *GAPDH* versus *RPL13* reinforced the variability of *GAPDH*, since we confirmed strong overexpression of *TP53* in metastasized tissues (as calculated with the use of *RPL13*) by IHC, as well as other authors found its increase expression in this type of samples [3, 35]. This point is another limitation of our study, since the RNA in situ hybridization (RNA ISH) should be applied in order to show the *TP53* mRNA content in cells instead of protein and confirm the results obtained by qPCR method. Since this method is currently unavailable to our team (i.e., RNA ISH requires different sample preparation [36]), we aimed to confirm the general *TP53* expression pattern in tumor-control-metastasized ccRCC samples by the means of IHC.

In comparison to GAPDH, GUSB and RPL13 proteins do not seem to be involved in ccRCC development, since GUSB is not engaged in intracellular metabolism, whereas RPL13 is highly expressed in the cytoplasm of all nucleated cells. Due to high stability, *GUSB* was noted as a suitable RG in lung [37] and ovarian [38] cancers, whereas *RPL13* was the best RG in Alzheimer’s disease [39] and in mesenchymal stem cell study [40]. We suppose that last reference is a key to the observed highest stability of *RPL13* in groups III–IV and may be caused by the high proportion of poorly developed (highly aggressive, Fuhrman’s stage 3–4) cells of tumor specimens in analyzed groups, which can be characterized by the increase deregulation or cellular machinery as compared to other specimens. Furthermore, the high differences in RG results between groups I–II and III–IV may be connected to the high proportion of metastatic tumor samples—from one fourth in group II to one half in group III, as well as to the introduction of metastatic tissue samples in group IV. We wanted to emphasize this factor, because not only the different type but also the proportion between the numbers of samples of different origin have strong influence on RG selection.

## Conclusions

For gene expression studies of mccRCC, we recommend to use a single RG (*GUSB* or *RPL13*) and to avoid the following RGs for normalization: *ACTB*, *B2M*, *GAPDH*, *TFRC*, and *UBC*.

## Electronic supplementary material

Below is the link to the electronic supplementary material.

ESM1 (DOCX 12 kb).

Supplementary Table 1 Expression stability ranking order of candidate reference genes divided into four groups (DOCX 21 kb).

Supplementary Table 2 Ct coefficient of variation percentage values for each gene or genes’ pair in groups’ analyzes (DOCX 16 kb).

Supplementary Figure 1 qPCR intra-run precision control experiment for *GUSB* assay. Amplification plot (left) shows fluorescence readings of 15 samples containing 10× diluted pooled cDNA from all ccRCC samples and 2 negative controls (*green lines*). Blue line—threshold (red – threshold value), Y axis – log fluorescence, X axis – cycle number. Right plot – melting curve of samples showing one specific PCR product. No amplification in negative controls – green lines close to X axis. Plots created in StepOne Software, ver. 2.2 (PPTX 967 kb).

Supplementary Figure 2 Summary expression data of each potential reference gene. Box (median—line, mean – “+”, upper and lower quartile) and whisker (10–90 % Ct values) plot of qPCR expression results (Y axis) for candidate reference genes (X axis) in alphabetical order. Shaded boxes—assays which passed D’ Agostino normality test. (GIF 26 kb)

High Resolution Image (TIFF 234 kb)
