# Supplementary material for: Identification of a suitable qPCR reference gene in metastatic clear cell renal cell carcinoma
Source: Tumour Biol. 2014 Sep 16;35(12):12473–87. doi: 10.1007/s13277-014-2566-9 (PMC4275580; doi:10.1007/s13277-014-2566-9)
Supplement: Supplementary file 1 — (DOCX 12 kb). [file 13277_2014_2566_MOESM1_ESM.docx]

Immunohistochemistry for TP53 protein

Formalin-fixed paraffin-embedded tissue sections (6 µm) from side tissues from 12 T/C/M samples were deparaffinized and hydrated through xylenes and graded alcohol series. After antigen retrieval using hot acidic citrate buffer (Epitope Retrieval Solution pH 6, Leica Biosystems Newcastle Ltd, NE12 8EW, United Kingdom) samples were blocked for endogenous peroxidase activity by using 3% hydrogen peroxide for 10 minutes. Sections were then incubated with 2,5% normal horse serum (ImmPRESS Anti-Rabbit Ig (peroxidase) Polymer Detection Kit, Vector Laboratories, Inc., Burlingame, CA 94010, USA) to block nonspecific binding of immunoglobulin. Immunohistochemical (IHC) staining was performed using TP53 rabbit anti-human polyclonal (N- terminus) antibody (1:50) (LifeSpan Biosciences, Inc., Seattle, WA 98121, USA). After 2 hours incubation with primary antibodies at room temperature, slides were washed in PBS and incubated with an appropriate secondary antibody (ImmPRESS Anti-Rabbit Ig (peroxidase) Polymer Detection Kit) for 30 min. Slides were rinsed in PBS and immunoreactive cells were visualized by addition of 3,3’-diaminobenzidine solution (DAB Peroxidase Substrate Kit, Vector Laboratories, Inc., Burlingame, CA 94010, USA) and counterstained with hematoxylin. Sections were then dehydrated, mounted in DPX mounting medium and viewed under a Nikon Eclipse E800 light microscope with Lucia G software. The specificity of the IHC staining was determined by a negative control, which was prepared under the same conditions as mentioned, replacing primary antibodies with 2,5% normal horse serum (ImmPRESS Anti-Rabbit Ig (peroxidase) Polymer Detection Kit).
