# Supplementary material for: Identification of a suitable qPCR reference gene in metastatic clear cell renal cell carcinoma
Source: Tumour Biol. 2014 Sep 16;35(12):12473–87. doi: 10.1007/s13277-014-2566-9 (PMC4275580; doi:10.1007/s13277-014-2566-9)
Supplement: Supplementary file 2 — Expression stability ranking order of candidate reference genes divided into four groups (DOCX 21 kb). [file 13277_2014_2566_MOESM2_ESM.docx]

Supplementary table 1. Expression stability ranking order of candidate reference genes divided into four groups.

| **GROUP I (all samples. 70 T + 70 C + 12 M)** | | | | | | | | | | | | | | | |
| --- | --- | --- | --- | --- | --- | --- | --- | --- | --- | --- | --- | --- | --- | --- | --- |
| Software used | Ranking order | | | | | | | | | | | | | | |
|  | Most stable | |  |  |  |  |  |  |  | |  |  |  | Least stable | |
|  | 1° | 2° | 3° | 4° | 5° | 6° | 7° | 8° | 9° | 10° | 11° | 12° | 13° | 14° | 15° |
| Delta CT [SD] | GUSB (1.74) | GAPDH (1.74) | IPO8 (1.76) | RPL13 (1.76) | RPL32 (1.79) | PPIA (1.82) | B2M (1.82) | ACTB (1.93) | RPLP0 (1.94) | HMBS (2.18) | HPRT1 (2.20) | TBP (2.21) | UBC (2.29) | TFRC (2.56) | PGK1 (2.61) |
| BestKeeper [SD + Pearson’s correlation coeficient] | GUSB (1.174) | RPL32 (1.225) | IPO8 (1.335) | RPL13 (1.485) | GAPDH (1.565) | PPIA (1.585) | UBC (1.652) | RPLP0 (1.667) | B2M (1.732) | HPRT1 (1.785) | TBP (1.789) | ACTB (1.801) | HMBS (1.845) | TFRC (1.914) | PGK1 (2.216) |
| Normfinder [stability value] | GUSB (0.881) | RPL32 (0.991) | IPO8 (1.019) | GAPDH (1.038) | RPL13 (1.049) | B2M (1.129) | PPIA (1.230) | RPLP0 (1.278) | ACTB (1.375) | HMBS (1.705) | HPRT1 (1.770) | UBC (1.772) | TBP (1.832) | TFRC (2.176) | PGK1 (2.306) |
| GeNorm [M stability value] | PPIA/ GAPDH (0.965) |  | RPL13 (1.036) | B2M (1.108) | ACTB (1.170) | IPO8 (1.223) | GUSB (1.303) | TBP (1.377) | HPRT1 (1.438) | RPL32 (1.530) | RPLP0 (1.621) | UBC (1.732) | HMBS (1.820) | TFRC (1.932) | PGK1 (2.023) |
| RefFinder summary ranking | **GUSB** (1.63) | **GAPDH** (2.51) | **IPO8** (3.57) | **RPL32** (3.76) | **RPL13** (3.94) | **PPIA** (3.98) | **B2M** (6.24) | **ACTB** (8.11) | **RPLP0** (8.92) | **HPRT1** (10.22) | **UBC** (10.70) | **TBP** (10.82) | **HMBS** (11.40) | **TFRC** (14.00) | **PGK1** (15.00) |

| **GROUP II (paired 70 T + 70 C)** | | | | | | | | | | | | | | | |
| --- | --- | --- | --- | --- | --- | --- | --- | --- | --- | --- | --- | --- | --- | --- | --- |
| Software used | Ranking order | | | | | | | | | | | | | | |
|  | Most stable | |  |  |  |  |  |  |  | |  |  |  | Least stable | |
|  | 1° | 2° | 3° | 4° | 5° | 6° | 7° | 8° | 9° | 10° | 11° | 12° | 13° | 14° | 15° |
| Delta CT | GAPDH (1.70) | GUSB (1.71) | IPO8 (1.71) | RPL13 (1.75) | RPL32 (1.77) | PPIA (1.80) | B2M (1.82) | RPLP0 (1.89) | ACTB (1.93) | HMBS (2.16) | HPRT1 (2.18) | TBP (2.22) | UBC (2.22) | TFRC (2.55) | PGK1 (2.59) |
| BestKeeper | GUSB (1.117) | IPO8 (1.176) | RPL32 (1.183) | RPL13 (1.405) | PPIA (1.434) | GAPDH (1.493) | UBC (1.589) | RPLP0 (1.639) | HPRT1 (1.656) | B2M (1.677) | TBP (1.712) | ACTB (1.730) | HMBS (1.782) | TFRC (1.876) | PGK1 (2.221) |
| Normfinder | GUSB (0.842) | IPO8 (0.950) | RPL32 (0.972) | GAPDH (1.003) | RPL13 (1.083) | B2M (1.157) | RPLP0 (1.215) | PPIA (1.221) | ACTB (1.388) | HMBS (1.689) | UBC (1.696) | HPRT1 (1.757) | TBP (1.851) | TFRC (2.189) | PGK1 (2.285) |
| GeNorm | PPIA/GAPDH (0.916) |  | RPL13 (0.973) | B2M (1.072) | ACTB (1.142) | IPO8 (1.197) | GUSB (1.284) | TBP (1.367) | HPRT1 (1.434) | RPL32 (1.522) | RPLP0 (1.603) | UBC (1.707) | HMBS (1.795) | TFRC (1.910) | PGK1 (2.000) |
| RefFinder summary ranking | **GUSB** (1.93) | **GAPDH** (2.21) | **IPO8** (2.91) | **PPIA** (3.94) | **RPL13** (3.94) | **RPL32** (4.61) | **B2M** (6.40) | **ACTB** (8.35) | **RPLP0** (8.38) | **HPRT1** (10.17) | **UBC** (10.47) | **TBP** (10.82) | **HMBS** (11.40) | **TFRC** (14.00) | **PGK1** (15.00) |

| **GROUP III (non-metastatic 35 T and mccRCC 35 T)** | | | | | | | | | | | | | | | |
| --- | --- | --- | --- | --- | --- | --- | --- | --- | --- | --- | --- | --- | --- | --- | --- |
| Software used | Ranking order | | | | | | | | | | | | | | |
|  | Most stable | |  |  |  |  |  |  |  | |  |  |  | Least stable | |
|  | 1° | 2° | 3° | 4° | 5° | 6° | 7° | 8° | 9° | 10° | 11° | 12° | 13° | 14° | 15° |
| Delta CT | RPL13 (1.64) | RPL32 (1.68) | RPLP0 (1.72) | GAPDH (1.72) | IPO8 (1.74) | B2M (1.74) | PPIA (1.75) | GUSB (1.76) | ACTB (2.03) | HMBS (2.13) | UBC (2.24) | TBP (2.25) | HPRT1 (2.48) | PGK1 (2.53) | TFRC (2.63) |
| BestKeeper | RPL13 (1.172) | GUSB (1.186) | RPL32 (1.200) | IPO8 (1.217) | GAPDH (1.420) | PPIA (1.432) | RPLP0 (1.450) | B2M (1.522) | TBP (1.659) | ACTB (1.784) | HMBS (1.862) | UBC (1.863) | TFRC (1.870) | HPRT1 (1.964) | PGK1 (2.237) |
| Normfinder | RPL32 (0.812) | RPL13 (0.827) | RPLP0 (0.919) | GUSB (0.972) | IPO8 (0.996) | B2M (0.998) | GAPDH (1.021) | PPIA (1.071) | ACTB (1.530) | HMBS (1.652) | UBC (1.726) | TBP (1.864) | HPRT1 (2.134) | PGK1 (2.203) | TFRC (2.293) |
| GeNorm | GAPDH/ RPL13 (0.893) |  | PPIA (0.930) | B2M (1.029) | IPO8 (1.097) | GUSB (1.231) | RPL32 (1.304) | RPLP0 (1.348) | ACTB (1.410) | TBP (1.504) | UBC (1.620) | HMBS (1.710) | HPRT1 (1.795) | PGK1 (1.905) | TFRC (2.002) |
| RefFinder summary ranking | **RPL13** (1.189) | **RPL32** (2.546) | **GAPDH** (3.44) | **GUSB** (4.427) | **IPO8** (4.729) | **RPLP0** (4.738) | **PPIA** (5.635) | **B2M** (5.826) | **ACTB** (9.24) | **TBP** (10.67) | **HMBS** (10.719) | **UBC** (11.242) | **HPRT1** (13.243) | **PGK1** (14.244) | **TFRC** (14.473) |

| **GROUP IV (matched 12 T + 12 C + 12 M)** | | | | | | | | | | | | | | | |
| --- | --- | --- | --- | --- | --- | --- | --- | --- | --- | --- | --- | --- | --- | --- | --- |
| Software used | Ranking order | | | | | | | | | | | | | | |
|  | Most stable | |  |  |  |  |  |  |  | |  |  |  | Least stable | |
|  | 1° | 2° | 3° | 4° | 5° | 6° | 7° | 8° | 9° | 10° | 11° | 12° | 13° | 14° | 15° |
| Delta CT | RPL13 (1.25) | PPIA (1.28) | TBP (1.30) | RPLP0 (1.31) | RPL32 (1.38) | PGK1 (1.40) | HPRT1 (1.41) | GAPDH (1.43) | IPO8 (1.43) | B2M (1.44) | HMBS (1.55) | ACTB (1.64) | GUSB (1.69) | TFRC (1.89) | UBC 2.11) |
| BestKeeper | RPL32 (1.630) | IPO8 (1.679) | HPRT1 (1.738) | TBP (1.745) | GUSB (1.846) | UBC (1.850) | PGK1 (1.873) | RPLP0 (1.889) | RPL13 (1.910) | GAPDH (1.948) | PPIA (1.948) | HMBS (1.988) | TFRC (2.056) | B2M (2.17) | ACTB (2.174) |
| Normfinder | RPL13 (0.632) | PPIA (0.683) | TBP (0.706) | RPLP0 (0.780) | RPL32 (0.875) | HPRT1 (0.906) | IPO8 (0.952) | PGK1 (0.967) | GAPDH (0.989) | B2M (0.996) | HMBS (1.144) | ACTB (1.257) | GUSB (1.351) | TFRC (1.599) | UBC (1.870) |
| GeNorm | RPL13/ RPLP0 (0.728) |  | PGK1 (0.892) | GAPDH (0.920) | PPIA (0.989) | TBP (1.025) | B2M (1.054) | RPL32 (1.124) | HPRT1 (1.174) | IPO8 (1.204) | HMBS (1.233) | ACTB (1.286) | GUSB (1.339) | TFRC (1.409) | UBC (1.502) |
| RefFinder summary ranking | **RPL13** (1.732) | **RPLP0** (3.364) | **RPL32** (3.761) | **TBP** (3.834) | **PPIA** (3.851) | **PGK1** (5.635) | **HPRT1** (5.803) | **IPO8** (5.958) | **GAPDH** (7.326) | **B2M** (9.950) | **GUSB** (10.238) | **HMBS** (11.242) | **UBC** (11.929) | **ACTB** (12.688) | **TFRC** (13.743) |
