# Supplementary material for: Identification of a suitable qPCR reference gene in metastatic clear cell renal cell carcinoma
Source: Tumour Biol. 2014 Sep 16;35(12):12473–87. doi: 10.1007/s13277-014-2566-9 (PMC4275580; doi:10.1007/s13277-014-2566-9)
Supplement: Supplementary file 3 — Ct coefficient of variation percentage values for each gene or genes’ pair in groups’ analyzes (DOCX 16 kb). [file 13277_2014_2566_MOESM3_ESM.docx]

**Supplementary table 2. Ct coefficient of variation percentage values for each gene or genes’ pair in groups’ analyzes.**

| Gene/ pair | Ct CV% | | | |
| --- | --- | --- | --- | --- |
|  | Group I | Group II | Group III | Group IV |
| ***ACTB*** | 12.24% | 12.02% | 13.28% | 13.65% |
| ***B2M*** | 12.76% | 12.30% | 11.68% | 15.81% |
| ***GAPDH*** | 11.33% | 10.86% | 10.87% | 13.16% |
| ***GUSB*** | **6.58%** | **6.19%** | 6.61% | 9.17% |
| ***HMBS*** | 8.68% | 8.39% | 8.89% | 10.59% |
| ***HPRT1*** | 9.33% | 8.71% | 10.05% | 8.98% |
| ***IPO8*** | 6.64% | 5.95% | 6.38% | 7.75% |
| ***PGK1*** | 14.24% | 14.34% | 14.99% | 14.56% |
| ***PPIA*** | 9.61% | 9.02% | 9.28% | 11.12% |
| ***RPL13*** | 8.61% | 8.27% | **7.29%** | **10.50%** |
| ***RPL32*** | 5.63% | 5.41% | 5.88% | 7.20% |
| ***RPLP0*** | 12.19% | 12.07% | 11.44% | 14.50% |
| ***TBP*** | 9.04% | 8.50% | 8.47% | 7.85% |
| ***TFRC*** | 9.05% | 8.92% | 8.91% | 10.23% |
| ***UBC*** | 6.50% | 6.34% | 7.11% | 7.01% |
| ***GAPDH + PPIA^$^*** | 10.19% | 9.67% |  |  |
| ***RPLP0 + TBP^$$^*** | 8.21% |  |  |  |
| ***PPIA + RPL13******^$$^*** |  | 8.36% |  |  |
| ***GAPDH + RPL13^$^*** |  |  | 8.80% |  |
| ***RPL13 + RPL32^$$^*** |  |  | 5.90% |  |
| ***RPL13 + RPLP0^$^*** |  |  |  | 12.34% |
| ***ACTB + TBP^$$^*** |  |  |  | 10.32% |

**$** RGs pair as selected by GeNorm or **$$** NormFinder for Groups I-IV. Ct data for RGs pairs was calculated as geometric mean of each assay. X – CtCV% value was not calculated for such pair due to selection for another group. Pale grey boxes – partial genes and summary pair for NormFinder selection; dark grey boxes – for GeNorm selection; average grey – genes for both GeNorm and NormFinder tools. Bold – CtCV% values for best single RGs for each group.
