# Supplementary figures and images for: Identification of a suitable qPCR reference gene in metastatic clear cell renal cell carcinoma
Source: Tumour Biol. 2014 Sep 16;35(12):12473–87. doi: 10.1007/s13277-014-2566-9 (PMC4275580; doi:10.1007/s13277-014-2566-9)

## Slide 1
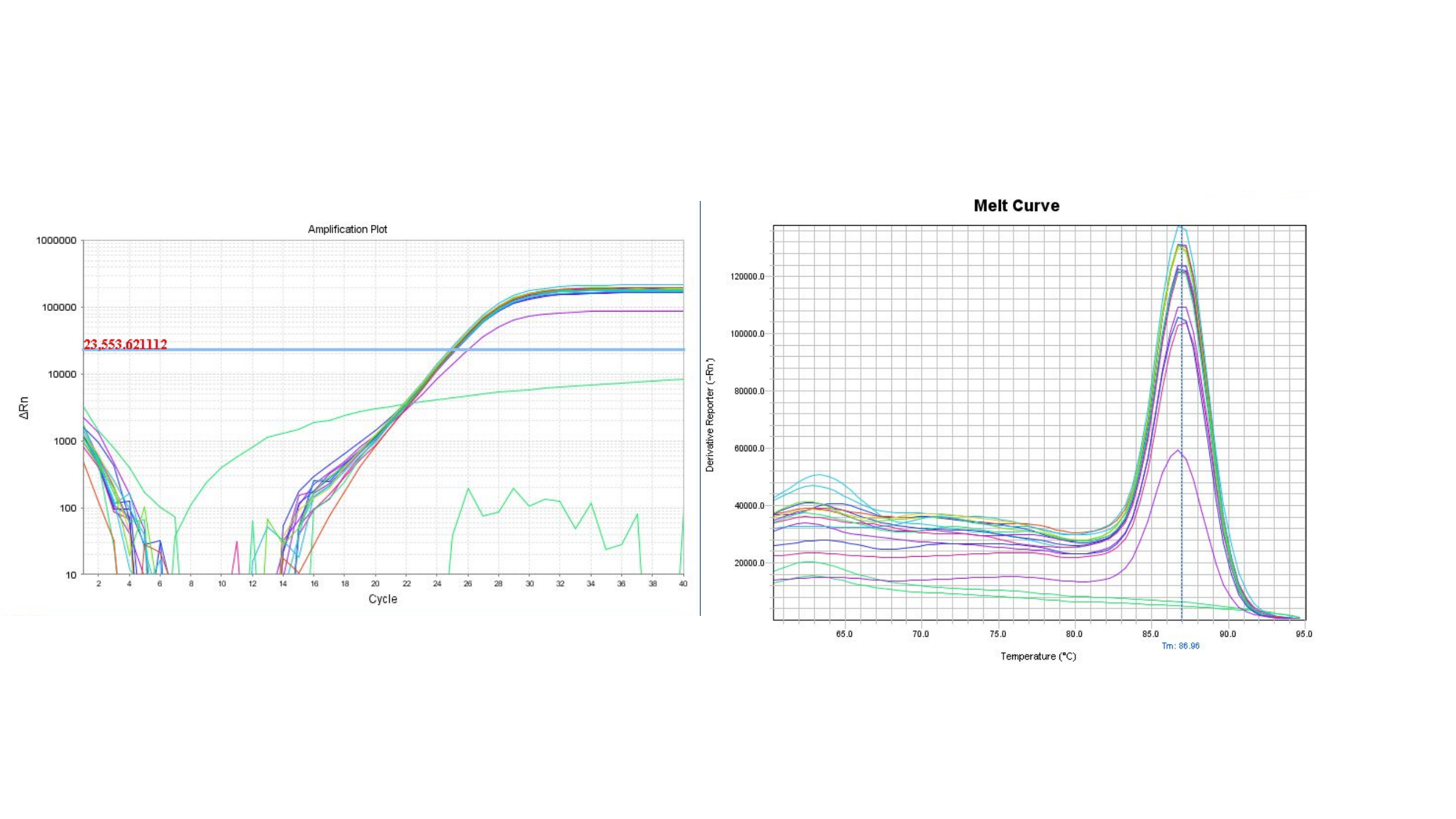

Supplement: Supplementary file 4 — qPCR intra-run precision control experiment for GUSB assay. Amplification plot (left) shows fluorescence readings of 15 samples containing 10× diluted pooled cDNA from all ccRCC samples and 2 negative controls (green lines). Blue line—threshold (red – threshold value), Y axis – log fluorescence, X axis – cycle number. Right plot – melting curve of samples showing one specific PCR product. No amplification in negative controls – green lines close to X axis. Plots created in StepOne Software, ver. 2.2 (PPTX 967 kb). [file 13277_2014_2566_MOESM4_ESM.pptx]

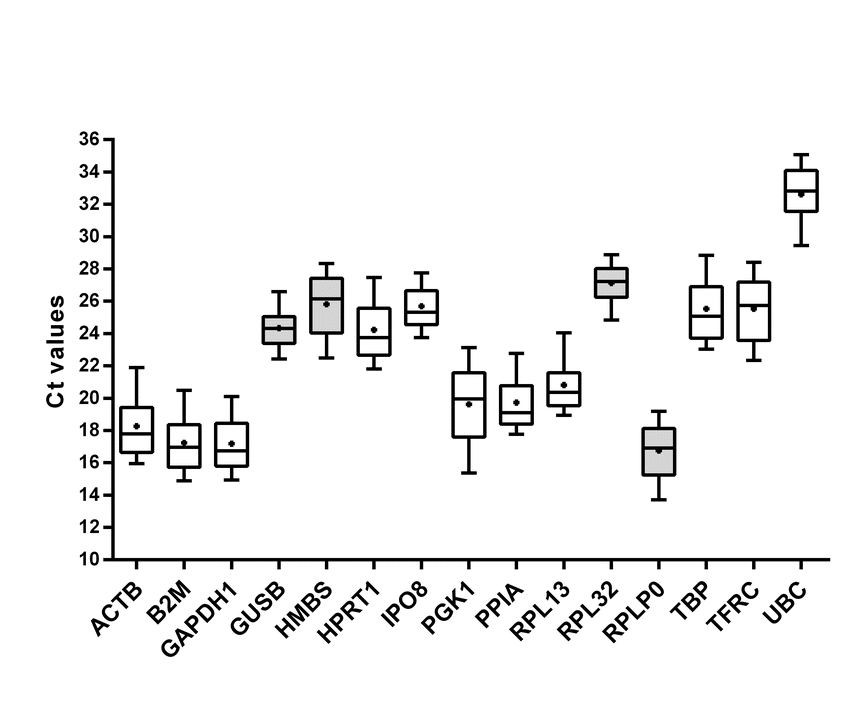

Supplement: Supplementary file 5 — Summary expression data of each potential reference gene. Box (median—line, mean – “+”, upper and lower quartile) and whisker (10–90 % Ct values) plot of qPCR expression results (Y axis) for candidate reference genes (X axis) in alphabetical order. Shaded boxes—assays which passed D’ Agostino normality test. (GIF 26 kb) [file 13277_2014_2566_Fig7_ESM.gif]

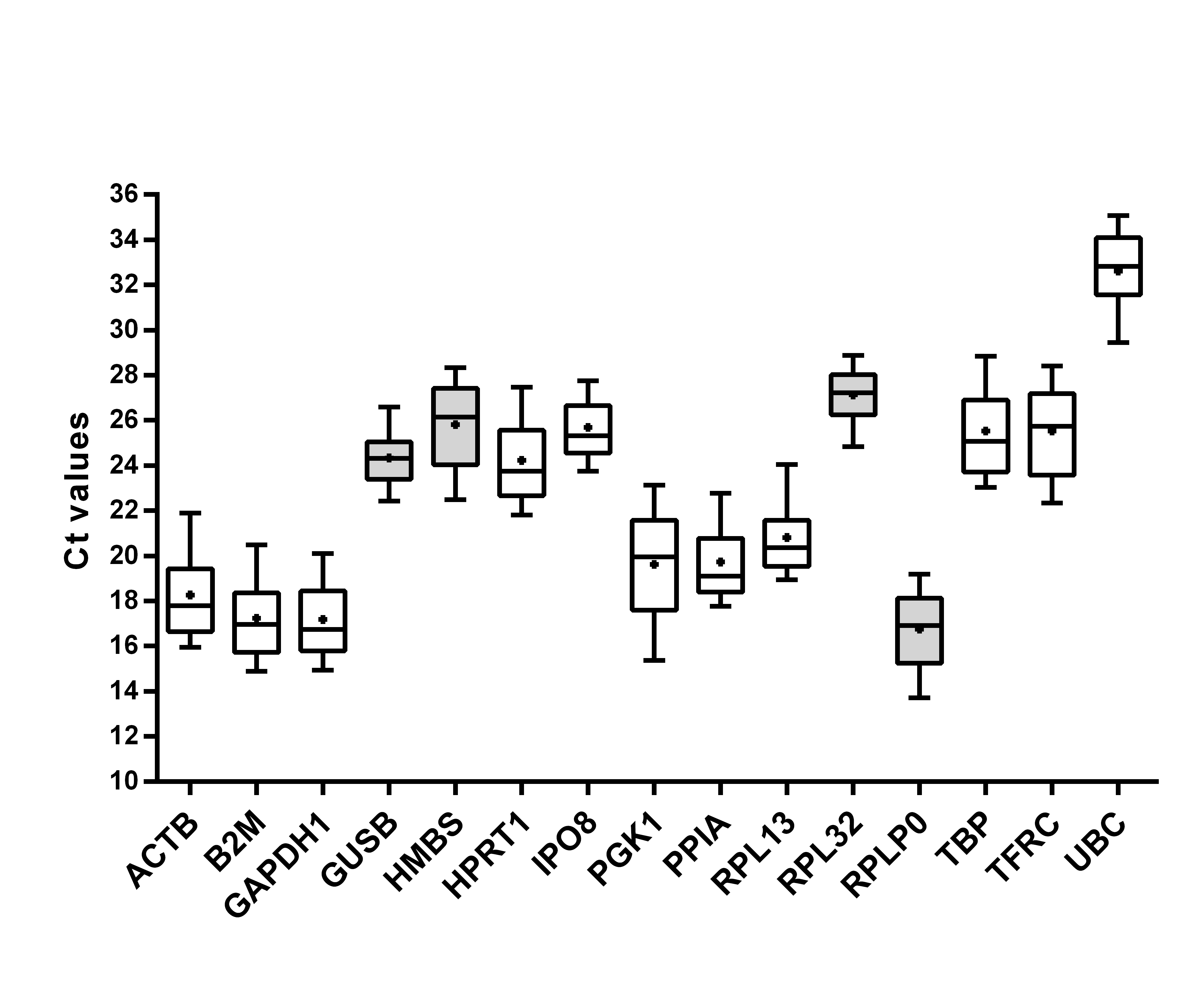

Supplement: Supplementary file 6 — High Resolution Image (TIFF 234 kb) [file 13277_2014_2566_MOESM5_ESM.tif]
